# Benzo[a]pyrene degradation by Kefir-derived microbiota: medium optimization and metabolic pathways

**DOI:** 10.3389/fmicb.2026.1873828

**Published:** 2026-07-15

**Authors:** Qing Wang, Yi Hu, Bei Zheng, Zhuonan Yang, Qianjing Lv, Rui Zhang, Yanan Qin

**Affiliations:** 1 Xinjiang Key Laboratory of Special Species Conservation and Regulatory Biology, College of Life Science, Xinjiang Normal University, Urumqi, Xinjiang, China; 2 Xinjiang Key Laboratory of Biological Resources and Genetic Engineering, College of Life Science & Technology, Xinjiang University, Urumqi, Xinjiang, China

**Keywords:** BaP degradation pathways, Kefir, meta-transcriptome, microbial communities, response surface optimization

## Abstract

Benzo[a]pyrene (BaP) is a highly carcinogenic and environmentally persistent polycyclic aromatic hydrocarbon (PAH) due to its low bioavailability and resistance to degradation. Microbial fermentation is a promising strategy to eliminate PAH residues in food processing, but the underlying mechanisms remain poorly characterized. Here, we optimized BaP biodegradation by Kefir-derived microorganisms using the Plackett-Burman design and response surface methodology (RSM). Under optimal conditions (12.50 mL of the trace element solution, 1.78 g/L Na_2_HPO_4_·2H_2_O, and 2.88 g/L NaCl), the microorganisms achieved a degradation efficiency of 51.78%. Meta-transcriptomic analysis postulated two key degradation pathways: (I) 3,4-dioxygenase (*α* and *β* subunits) catalyzes the initial dioxygenation to produce phenanthrene, and (II) a fluorene-mediated transformation facilitates extensive degradation via the salicylic and phthalic acid pathways. These findings indicate the potential of Kefir-derived microbiota to detoxify BaP in food systems.

## Introduction

As a class of persistent organic pollutants (POPs) characterized by strong carcinogenicity, teratogenicity, and mutagenicity, polycyclic aromatic hydrocarbons (PAHs) are widely present in environmental media (the atmosphere, water, and soil) and food (Premnath et al., 2021). They are particularly enriched in food products through thermal processing such as smoking and barbecuing, posing a serious threat to human health (Wang and Fang, 2025; Du et al., 2025). Benzo[a]pyrene (BaP) is the most prominent representative of this pollutant class. Designated as a Group I carcinogen by the International Agency for Research on Cancer (IARC) and listed among the top ten priority pollutants by the US Environmental Protection Agency (USEPA) (Chen et al., 2023), chronic exposure to it is associated with chest pain, coughing, dermatitis, respiratory problems, and immune dysfunction (Tartaglione et al., 2023). Conventional technologies for removing PAHs from contaminated environments include traditional physical processes like adsorption and volatilization (Queiroz et al., 2022), as well as advanced chemical treatments such as ozonation, direct photolysis, hydrogen peroxide treatment, and Fenton and photo-Fenton processes (Rubio-Clemente et al., 2014). However, these methods are often costly and do not guarantee complete mineralization of the compounds. As a more sustainable alternative, biodegradation relies on microorganisms such as bacteria, fungi, and algae to break the chemical bonds present in these pollutants (Ismail et al., 2022), offering an efficient strategy to eliminate PAHs, particularly BaP residues, from the environment and food products.

Kefir is a natural fermented dairy beverage obtained by fermenting milk with kefir starter grains. As a natural complex microbial ecosystem, the kefir microbial community synthesizes a variety of extracellular enzymes during metabolism, which can convert BaP into low or non-toxic metabolites through epoxidation, hydroxylation and other reactions (Duo et al., 2025). Previous studies have shown that kefir possesses remarkable microbial diversity, comprising bacteria such as *Bacillus*, *Lactobacillus*, *Klebsiella*, *Enterobacter*, *Acinetobacter*, and *Enterococcus*, alongside fungi including *Saccharomyces*, *Kazachstania*, *Kluyveromyces*, and *Pichia*, occupy a prominent position (Wang and Guo, 2023). Notably, *Lactobacillus* and *Debaryomyces hansenii* have been reported to degrade PAHs such as BaP, providing a rationale for using kefir as a resource pool to identify high-quality PAH-degrading microbes (Książek-Trela et al., 2024). Sultana et al. (2021) isolated a BaP-tolerant probiotic strain, *Bacillus velezensis* PMC10, from fermented foods, which achieved a high degradation efficiency of 51.32%. Furthermore, *B. velezensis* is not only non-cytotoxic but also exhibits significant anti-inflammatory and antioxidant activities, endowing it with potential as a probiotic for the degradation or detoxification of PAHs in the human gut or skin, thereby exerting a protective effect on human cells during its application.

The composition of the culture medium is a key factor influencing BaP degradation efficiency by kefir microbiota. This influence is exerted not only by providing basal nutrition for microbial growth but also by modulating physiological metabolism, community structure, and interspecies interactions through various mechanisms, ultimately determining degradation performance. Trace elements in the culture medium serve as enzyme cofactors, catalyzing the oxygenation and cleavage reactions of the BaP ring, thereby substantially enhancing degradation (Guzik et al., 2013). Research has demonstrated that in the protocatechuate 3,4-dioxygenase system, the inclusion of iron ions increases the relative enzyme activity of free and immobilized extracts to 16 and 99%, respectively, whereas copper ions (Cu^2+^) exhibit comparable activation effects (Silva et al., 2013). Conversely, high concentrations of phosphate or sodium chloride in the medium can inhibit cofactor availability, thereby reducing the microbial degradation capacity (Zafar et al., 2010). Given that medium components profoundly influence these physiological and biochemical processes, it is crucial to understand the molecular mechanisms underlying their regulatory effects. Meta-transcriptomics provides a powerful approach to address this gap by dynamically monitoring transcriptional gene expression differences, it captures the temporal response patterns of bacterial community functions and the role of key enzymes in aromatic ring cleavage (Wang S. et al., 2025), offering a new perspective for systematically elucidating the degradation process. Therefore, improving BaP degradation efficiency mediated by kefir microbiota requires further investigation into the effects of mineral medium components on both microbial community structure and degradation pathways. Optimizing culture conditions is essential to fully harness the degradation potential of kefir microbiota.

In this study, we optimized the mineral medium composition to maximize the degradation efficiency of BaP by kefir microbiota and to identify the most suitable formulation. Concurrently, we comprehensively investigated the transcriptional profiles and molecular mechanisms underlying microbial BaP degradation in this optimized medium. A key innovation of this work is the fusion of traditional medium optimization with multi-omics technology, aimed at overcoming the efficiency limitations of single-strain BaP degradation. This dual approach not only facilitates the engineering of microbiota to combat food-borne PAHs but also serves as a valuable reference for constructing synthetic microbial communities capable of degrading complex pollutants.

## Materials and methods

### Chemicals and mediums

Benzo[a]pyrene (analytically pure, >99%), acetone, dichloromethane, methanol (all chromatographically pure, >99%) purchased from Beijing Dingguo Changsheng Biotechnology Co Ltd. (Beijing, China). Given that the desired BaP concentrations greatly surpassed its aqueous solubility, a stock solution was prepared by dissolving 1.0 g of BaP in 1,000 mL of sterilized acetone (1.0 g/L) and maintained at −20 °C until required. The minimal-salt medium (MSM) was prepared by dissolving 0.2 g/L MgSO_4_·6H_2_O, 0.5 g/L (NH_4_)_2_SO_4_, 1.0 g/L NH_4_NO_3_, 0.5 g/L KH_2_PO_4_·H_2_O, 1.5 g/L K_2_HPO_4_, and 0.5 g/L NaCl in distilled water.

### Microorganisms and culture conditions

Kefir grains were sourced from an ethnic minority family in the Kashgar region of Xinjiang. For activation, 2% (w/v) of the kefir grains were inoculated into whole-fat pasteurized cow milk (3.5% milkfat) and incubated at 25 °C for 24 h in autoclaved glass jars. Following fermentation, 20 mL of the fermented milk was centrifuged at 4,000 rpm for 5 min. The entire supernatant was then transferred to a new tube and centrifuged at 12,000 rpm for 10 min to completely pellet the microbes. Finally, 200 μL of PBS buffer was added to the pellet, which was then vortexed until the microbial biomass was completely resuspended, yielding a Kefir microbial suspension.

The degradation capacity of the Kefir microbiota was evaluated using the shake-flask method. Ten milliliters of Kefir microbial suspension was inoculated into 90 mL of optimized liquid MSM containing 20 mg/L BaP. The uninoculated control was prepared without the addition of the Kefir microbial suspension. The cultures were incubated at 37 °C in an orbital shaker at 140 rpm for 2 days. Each set of experiments was performed at least in triplicate.

### Extraction and quantification of BaP

At 0, 12, 24, 36, and 48 h, 1.0 mL aliquots of the culture were withdrawn and extracted with an equal volume of dichloromethane. The organic phase was collected, the extraction was repeated twice, and the combined extracts were filtered through a 0.22 μm organic phase filter membrane. The extracts were analyzed using high-performance liquid chromatography (HPLC), equipped with C18 Diamosil™ reverse-phase column (4.6 mm × 250 mm, particle size 5 μm). The injection volume was 20 μL, and the flow rate was maintained at 1.0 mL/min. The column temperature was set to 37 °C, and the mobile phase consisted of methanol:water (100:0, v/v). The detection wavelength was 245 nm, and the retention time was 10 min. The degradation rate was calculated using the following equation:


$$\mathrm{Rd}=(C_{0}-C_{n})/C_{0}\times 100\%$$

where, Rd (%) represents degradation rate, C_0_ (mg/L) is the initial concentration of BaP and C_n_ (mg/L) is the residual concentration of BaP after incubation for n hours.

### Plackett-Burman design (PBD)

PBD was used to screen the mineral media components to identify the components that significantly affect the biodegradation of BaP by Kefir microorganisms (Plackett and Burman, 1946). Analyses were performed using the statistical software package Design-Exper 10. The nine variables screened consisted of various mineral media components (denoted as A to I, where A is KH_2_PO_4_, B is K_2_HPO_4_, C is Na_2_HPO_4_ 2H_2_O, D is NaCl, E is NH_4_Cl, F is MgSO_4_ 6H_2_O, G is MnSO_4_, H is FeCl_3_, and I is Trace Elements Solution SL-4). Each variable was set to two levels: high (+1) and low (−1), where the concentration corresponding to the each level was determined based on the range of concentrations reported in the literature for the bacterial degradation of heterocyclic PAHs in mineral media (Deriase et al., 2012; Wang and Fang, 2025). Supplementary Table S1 lists the nine mineral medium fractions and the corresponding code names and their high and low concentrations for variables A through I. Supplementary Table S2 presents PB design matrix showing the media compositions used in different experimental runs and the corresponding BaP degradation rates.

### Method of steepest ascent (SAM)

To rapidly approach the optimal region, based on the effect analysis of the PB experimental results and combined with the practical needs of the test, the steepest climbing test paths were designed for the three significant factors according to their positive and negative effects, including the step size and direction of change of each factor, to approach the maximum response region as quickly as possible and to determine the center point for the subsequent response surface optimization experiment.

### Response surface models (RSM)

The main and interaction effects of the factors Na_2_HPO_4_ 2H_2_O, NaCl, and trace elements solution concentration on the degradation of BaP were optimized using RSM and a three-variable Box–Behnken design performed in the statistical analysis software (Design-Expert 10). The study consisted of 17 experimental runs with five replications at the center point. All experimental runs were performed in triplicate as independent biological replicates. An uninoculated minimal-salt medium (MSM) containing BaP was included as a negative control to account for abiotic loss. Based on the previous studies, three key factors and their optimal ranges were selected for this experiment: concentration of Na_2_HPO_4_ 2H_2_O (1–3 g/L), concentration of NaCl (1–5 g/L) and trace elements solution (10–20 mL/L) (Supplementary Table S3). The collected data were analyzed using response surface methodology with BaP degradation efficiency as the response value.

### Meta-transcriptomics RNA extraction and shotgun sequencing

Samples collected at degradation time points were designated as QMSM0, OMSM12, OMSM24, OMSM36, and OMSM48, respectively, with three independent biological replicates for each time point. These samples were sent to Shanghai Personal Biotechnology Co., Ltd. (Shanghai, China) for meta-transcriptome sequencing. Total RNA was extracted using the RNA PowerSoil® Total RNA Isolation Kit (12866–25) (MoBio, USA) according to the manufacturer’s instructions. The extracted RNA was assessed for quality and quantity using 1.5% agarose gel electrophoresis and a UV spectrophotometer. Meanwhile, RNA integrity (RIN > = 5.5) was assessed using an Agilent2100 (Agilent, USA). After ribosomal RNA removal, the RNA TruSeq Stranded mRNA LT Sample Prep Kit (Illumina, USA) was used for reverse transcription and meta-transcriptome shotgun sequencing library construction.

For data normalization, raw read counts were processed using DEGseq (version 1.26.0) with its built-in normalization method to account for differences in library size. Additionally, Transcripts Per Million (TPM) was calculated to quantify relative gene expression levels. Differentially expressed genes (DEGs) were identified with the conditions set at |log_2_ (fold change) | > 1 and *p* < 0.05. Gene Ontology (GO) enrichment analyses were conducted to explore the roles of these DEGs, with GO terms having a *p* < 0.05 considered significantly enriched by mapping each DEG to the GO database.^1^ Additionally, KEGG pathway analysis was employed to annotate biological pathway responses, where pathways with *p* < 0.05 were deemed significantly enriched. All analyses were conducted using Shanghai Personal Biotechnology Ltd.’s online Cloud Platform^2^. The data reported in this paper have been deposited in OMIX (China National Bioinformatics Center/Omics Raw Data Archive) (https://ngdc.cncb.ac.cn/gsa/browse/CRA041797: GSA accession number CRA041797).

### Statistical analysis

Sample differences and the significance of each result were analyzed using analysis of variance (ANOVA) in SPSS 27, with a *p*-value of less than 0.05 deemed statistically significant. Graphical representation was performed using Origin 24 and Adobe Illustrator 2024. All experimental assays were replicated three times to ensure reliability.

## Results and discussion

### Influence of medium composition on the degradation of BaP

#### Plackett-Burman design

Plackett-Burman is a two-level test method that allows rapid screening of significant influencing factors from a large number of variables with fewer experiments, and is commonly used to screen mineral media components. The Plackett-Burman (PB) method was used to screen the main factors affecting the degradation rate, and the data were regressed and analyzed using the BaP degradation rate (Y) as the response value. The ANOVA results in Table 1 showed that the model was significant and the fit was successful. The model equation including all terms regardless of their significance is shown in Equation 1.


$$\begin{array}{l} Y\;\;(\%)=26.26+3.01A+3.69B-6.30C-5.62D+\\ \;4.90E+1.98F-0.55G-0.20H+7.11I \end{array}$$ (1)

**Table 1:** Variance analysis for Plackett-Burman experiment.

variable	Sum of squares	Degrees of freedom	Mean square	*F*-value	*p*-value	Significance
Model	2074.22	9	230.47	33.89	0.0290	**
A	108.42	1	108.42	15.94	0.0574	
B	163.32	1	163.32	24.02	0.0392	**
C	476.91	1	476.91	70.13	0.0140	**
D	379.58	1	379.58	55.82	0.0174	**
E	288.41	1	288.41	42.41	0.0228	**
F	47.01	1	47.01	6.91	0.1193	
G	3.60	1	3.60	0.53	0.5427	
H	0.50	1	0.50	0.074	0.8117	
I	606.48	1	606.48	89.18	0.0110	**
Residual	13.60	2	6.80			
Total	2087.83	11				

R^2^ = 0.9935; R_adj_^2^ = 0.9642. *represents p < 0.05, and **represents *p* < 0.01.

Falagán et al. (2024) demonstrated that switching the culture medium from nutrient-supplemented conditions to ammonium- and phosphate-free M9 medium shifted community dominance, with *Acidithiobacillus* decreasing from 82 to 91 to 19% and *Thiobacillus* rising to 77%. Consequently, optimizing these factors could not only modulate microbial metabolism but also facilitate the selection and enrichment of specific microbial consortia with enhanced degradation capabilities, thereby improving the overall efficiency of PAHs degradation. Our statistical analysis showed that I (trace elements solution), C (Na_2_HPO_4_ 2H_2_O) and D (NaCl) were the key factors in the mineral medium compositions affecting the BaP degradation by Kefir-derived microorganisms. Therefore, BaP degradation could be maximized by adjusting these factors. The important role of trace elements on degradation of oil and PAHs have been demonstrated in several earlier studies (Deriase et al., 2012; Liu et al., 2025; Zhong et al., 2007). Trace elements play a crucial role in the growth (Gao et al., 2018), activity, and physiological functions of microorganisms. They are key cofactors in redox reactions, structural components of enzymes, and electron carriers. In contrast, high concentrations of phosphate or NaCl may inhibit the availability of cofactors, which in turn affects microbial degradation. This inhibition of cofactor availability may be occur by interfering with microbial metabolic pathways or enzyme activities.

#### Steepest ascent method (SAM)

The path of steepest ascent was determined based on actual conditions. Factor I (trace elements solution) had a positive impact, while the other two factors C (Na_2_HPO_4_ 2H_2_O) and D (NaCl) had negative impacts on BaP degradation. Therefore, mineral media were designed as shown in Table 2, where I was increased from the starting point while C and D were decreased from the starting point and kept at the level of the center point in the PBD space at regular intervals. The results of test 3 showed that the maximum the BaP degradation rate was achieved by increasing the concentration of I (trace elements solution) and reducing the concentrations of C (Na_2_HPO_4_ 2H_2_O) and D (NaCl). Therefore, test 3 was chosen as the central point for the next phase of response surface optimization.

**Table 2:** Experimental design and results of the steepest ascent method.

Code	Trace elements (mL) (I)	Na_2_HPO_4_ 2H_2_O (g/L) (C)	NaCl (g/L) (D)	Degradation rate (%)
1	5	4	5	28.44 ± 0.56
2	10	3	4	37.87 ± 0.87
3	15	2	3	46.23 ± 0.62
4	20	1	2	34.09 ± 0.49
5	25	0	1	26.32 ± 0.76

### Box–Behnken design (BBD) using response surface method (RSM)

The experimental design and the response of dependent variables for the degradation efficiency of BaP (Y) are presented in Supplementary Table S3. Data from Table S3 were processed by response surface regression procedure using Design-Expert 10 software, and results were obtained by fitting the data with the second-order polynomial model shown in Equation 2.


$$\begin{array}{l} Y(\%)=49.78-5.09I-3.07C-0.35D+1.85\mathrm{IC}+\\ \;2.12\mathrm{ID}-0.15\mathrm{CD}-5.91I^{2}-8.97C^{2}-10.72D^{2}\; \end{array}$$ (2)

**Figure 1:**
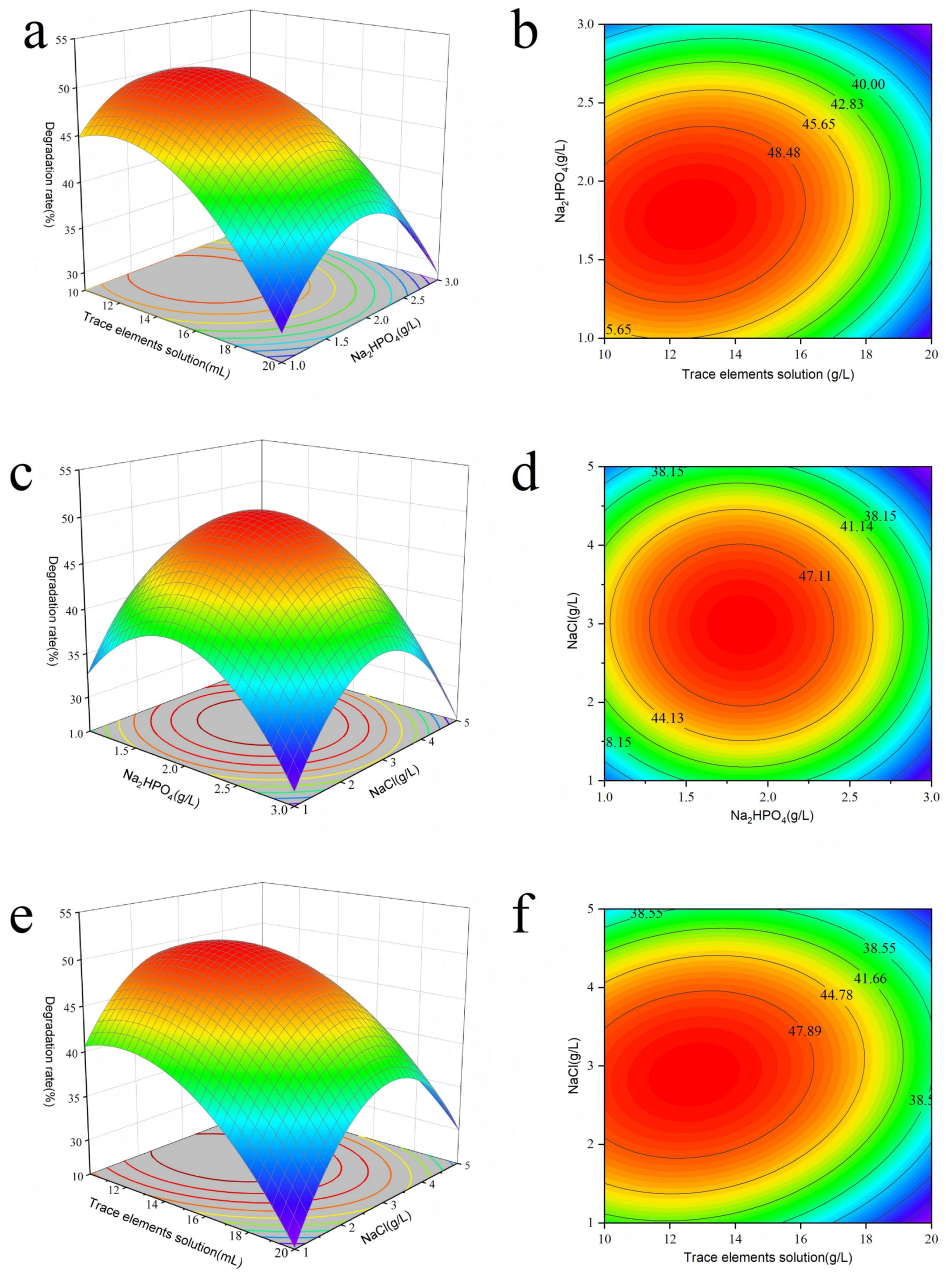
Response surface plots showing the interaction effects of factors on BaP degradation.3D response surfaces **(a,c,e)** and contour plots **(b,d,f)** illustrating the BaP degradation as influenced by trace elements solution, disodium hydrogen phosphate, and sodium chloride.

The results of analysis of variance (ANOVA) (Table 3) showed that the model was significant at *p* = 0.0002; the lack-of-fit term *p* > 0.05 was not significant, indicating that unmeasured factors had little influence on the experiment. The coefficient of determination, R^2^ was 0.9250, indicating that the model could accurately reflect the actual relationship between the degradation efficiency of BaP and the component factors. The response surface plot depicting the interaction of the three factors at three levels was shown in Figure 1. The established mathematical model was optimized and analyzed to obtain the optimal medium composition for degrading BaP: 12.50 mL of trace elements, 1.78 g/L of disodium hydrogen phosphate, and 2.88 g/L of sodium chloride, for which the model predicted a maximum degradation rate of 51.34%. The validation test was repeated three times under the optimal conditions, and the degradation efficiency of BaP reached 51.78% after 48 h. This observed value was very close to the predicted value, which proved the reliability of the model. The relative error between the actual measured degradation rate (51.78%) and the predicted value was small, indicating that the proposed model has good prediction accuracy and reliability.

**Table 3:** ANOVA results of the quadratic model.

Variable	Sum of squares	Degrees of freedom	Mean square	*F*-value	*p*-value	Significance
Model	1391.28	9	154.59	22.91	0.0002	**
I	207.67	1	207.67	30.78	0.0009	**
C	75.58	1	75.58	11.20	0.0123	*
D	0.99	1	0.99	0.15	0.7135	
IC	13.69	1	13.69	2.03	0.1973	
ID	17.98	1	17.98	2.66	0.1466	
CD	0.093	1	0.093	0.014	0.9098	
I^2^	147.13	1	147.13	21.81	0.0023	**
C^2^	338.69	1	338.69	50.20	0.0002	**
D^2^	483.75	1	483.75	71.70	<0.0001	**
Residual	47.23	7	6.75			
Loss of fit	21.80	3	7.27	1.14	0.4332	
Pure error	25.43	4	6.36			
Total	1438.51	16				

R^2^ = 0.9250; R^2^adj = 0.9299. *represents p < 0.05, and **represents *p* < 0.01.

RSM has been extensively utilized not only to optimize degradation conditions but also to decipher the complex interactive effects among multiple variables that govern PAH biodegradation. For example, Lv et al. (2025) applied RSM to optimize medium pH, temperature, initial substrate concentration, and inoculum size, revealing how the interplay between these factors dictates the physiological state and enzymatic activity of strain MA-4, thereby significantly enhancing BaP degradation efficiency. Similarly, Zou et al. (2023) isolated *Acinetobacter* XS-4 from coking wastewater, demonstrating that navigating the interactive effects of environmental factors via RSM was crucial for alleviating substrate toxicity and optimizing metabolic activity, which allowed the strain to degrade 62.73% of BaP (10 mg/L) within 60 h under optimal conditions. In the present study, RSM optimization of medium components enabled the kefir microbial consortium—a complex symbiotic system derived from traditional fermented dairy products—to achieve a degradation efficiency of 51.78% for 20 mg/L BaP in 48 h. Unlike single-strain systems, the interaction effects identified by RSM in the kefir system reflect the intricate nutritional cross-talk that drives synergistic interspecies metabolism; optimizing these components simultaneously sustains the community structure and co-activates the catabolic pathways necessary for ring cleavage. Parallel to this, Liu et al. (2024) demonstrated that repeatedly acclimating and enriching activated sludge under 40 mg/L high molecular weight pyrene stress selectively enriched degraders like *Thauera*, and after community optimization, pyrene removal reached 92.4% in 54 h; this microbial community optimization increased the activities of initial dioxygenase (NDO) and catechol 2,3-dioxygenase (C23O), and the synergy between community structure and key enzymes significantly enhanced aromatics mineralization. Meanwhile, in our study, RSM optimization results also indicated that the kefir microbial consortium achieved significant degradation of high molecular weight PAH BaP in a relatively short time. Consequently, the significant degradation ability of BaP exhibited by this synergistic microbial system is comparable to that of specialized strains in various environments.

### Microorganisms and pathways involved in BaP degradation

#### Response of microbial communities in BaP degradation under optimized medium compositions

**Figure 2:**
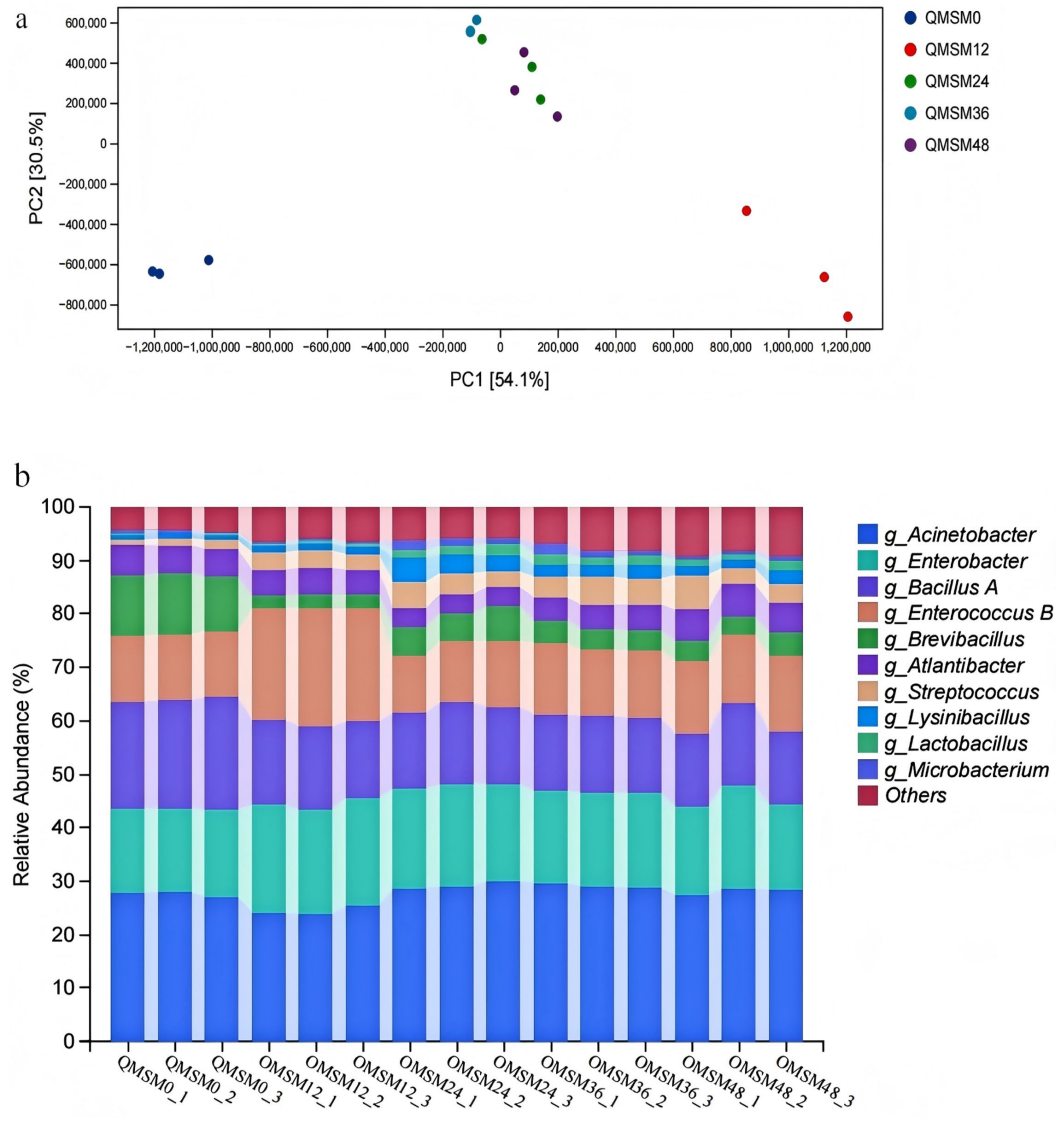
Microbial community analysis of kefir samples. **(a)** PCA plot. PCA illustrates the degree of separation among all groups; **(b)** Microbial community bar plot at the genus level. Relative abundance of microbial communities at the genus levels in kefir samples.

Meta-transcriptomics techniques were used to investigate the enrichment culture of Kefir samples at different time periods, and the results showed that the Kefir microbiota in the modified medium under BaP stress exhibited unique metabolic adaptations at various stages, leading to differences in the community structure. As shown in Figure 2a, the results of principal component analysis (PCA) indicated that the greatest difference in community structure was observed between 0 h (QMSM0) and 12 h (OMSM12) of incubation, whereas the samples at 24 h (OMSM24), 36 h (OMSM36) and 48 h (OMSM48) were highly similar in terms of their community composition. This dynamic change reflected the establishment of metabolic dominance through rapid response of functional genes in the early stage of degradation (0–12 h), while the community stabilized through functional redundancy and synergistic metabolism in the middle and late stages (24–48 h) as intermediate metabolites accumulated. The PCA results not only verified the temporal response pattern of microbial communities to BaP stress, but also provided an important basis for analyzing the coupled relationship between the structure and function of the bacterial communities in complex environments.

Given that fungal taxa were less significant than bacterial taxa in the diversity results, the subsequent analysis focuses on the bacterial community. Among the identified bacterial phyla (Supplementary Figure S1), it was found that the microbial community structure of Kefir under BaP stress at different enrichment incubation times was relatively similar, being mainly dominated by Firmicutes and Proteobacteria. Firmicutes was the dominant community for BaP degradation in kefir, and its core genera: *Lactobacillus*, *Streptococcus* and *Bacillus* directly drove the degradation process; Proteobacteria played a supporting role through Neisseriaceae (Duo et al., 2025; Wang and Fang, 2025). Figure 2b showed the changes in the relative abundances of the top twenty genera of Kefir samples in enrichment culture at different time periods (0 h, 12 h, 24 h, 36 h, 48 h), among them, *Acinetobacter*, *Enterobacter*, *Bacillus A*, *Enterococcus B* and *Brevibacillu* were the dominant genera. The relative abundance of *Enterobacter* and *Enterococcus B* increased by 3.94 and 9.19%, respectively, from 0 to 12 h during the initial degradation period, indicating their strong initial degradation ability for BaP. However, *Acinetobacter*, *Bacillus A* and *Brevibacillu* decreased in abundance at the initial stage of degradation due to their inability to tolerate BaP toxicity or poor environmental adaptation. During the mid-degradation phase (12–24 h), the relative abundances of *Acinetobacter* and *Brevibacillu* began to increase, suggesting their involvement in the degradation of intermediate metabolites such as phthalic acid and salicylic acid. This finding is consistent with the results reported by Zou et al. (2023), which indicated the presence of salicylic acid and phthalic acid pathways during the BaP degradation process by *Acinetobacter* XS-4. Microbial community stabilized after 24 h. *Acinetobacter*, *Enterococcus* and *Bacillus* constituted the core degrading consortium, and the significant degradation of BaP was achieved through synergistic effects.

#### Gene response and functional annotation of kefir microorganisms to BaP stress

**Figure 3:**
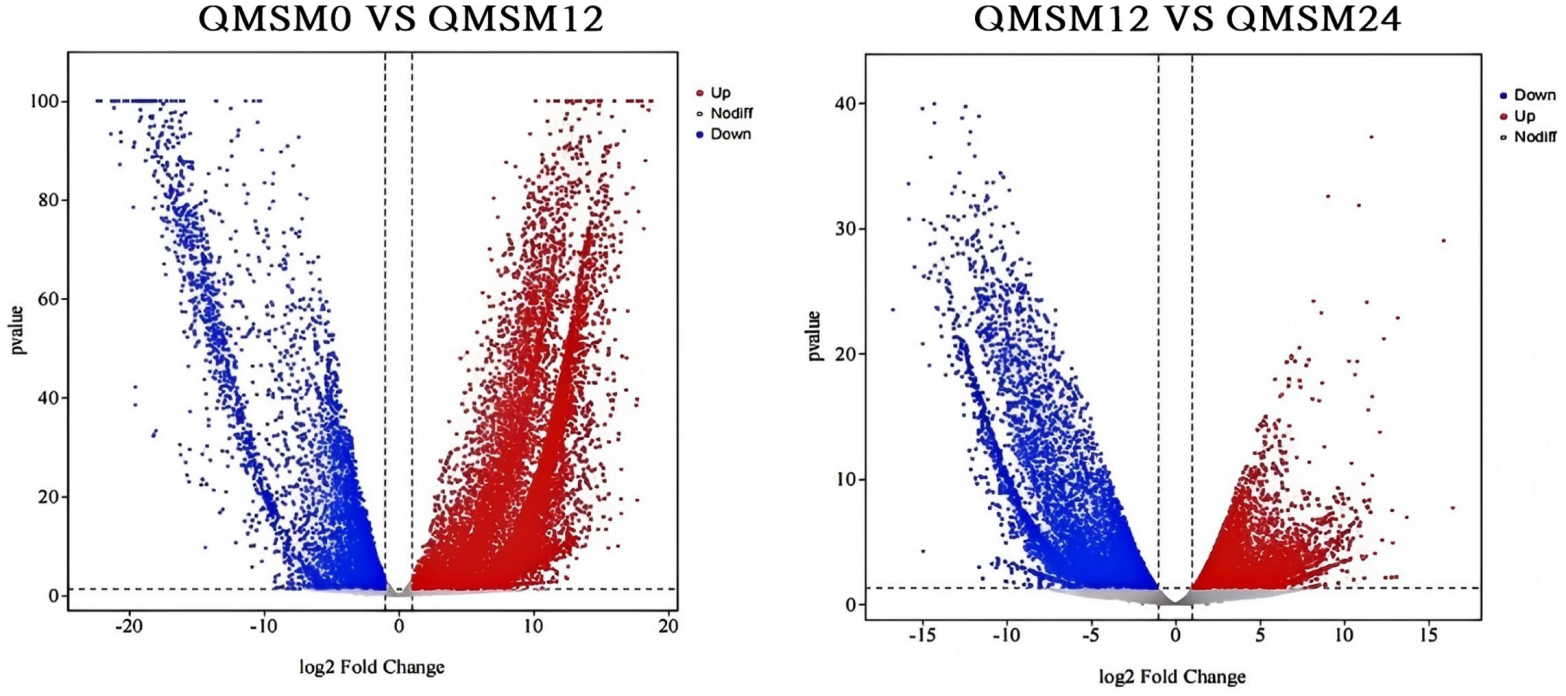
Volcanic plot of differentially expressed genes at different degradation stages. The horizontal axis of the volcano plot represents the fold change (log_2_FC) of differentially expressed genes between the compared groups, while the vertical axis represents the statistical significance (*p*-value) higher values correspond to more significant expression differences. Both axes are logarithmically scaled. Blue dots indicate downregulated genes whereas red dots indicate upregulated genes.

To elucidate the response mechanism of kefir samples in optimized medium under BaP stress, we obtained basic information on differential gene expression (DEG) during different periods of BaP degradation by kefir through quantitative analysis of gene expression and fold change analysis. A total of 22,600 DEGs, (18,517 up-regulated and 4,083 down-regulated genes) were observed in the comparison of 0 h vs. 12 h; and 13,773 DEGs, (6,517 up-regulated and 7,256 down-regulated genes) were observed in 12 h vs. 24 h (Figure 3).

During the initial stage of BaP degradation (0–12 h), the dioxygenase gene (*nidA*) was activated, which potentially drove the aromatic ring opening reaction. At the same time, the aldehyde dehydrogenase (*nidD*) gene and the salicylaldehyde dehydrogenase (*nahF*) gene showed high expressi on levels, which suggested a potential promotion of the conversion of intermediate products to benzoic acid. When the degradation proceeded to the late stage (24–48 h), genes related to the tricarboxylic acid cycle (TCA) were significantly enriched to complete the metabolic transformation of small molecules (Supplementary Figure S2). This division of labor and metabolism based on the transcriptional response of key genes in different periods enables the microbial community to complete the degradation task of BaP in an efficient manner. Combined with the change of microbial community structure, it was found that *Enterobacter* played a major role in the early process of BaP degradation. *Acinetobacter* played a key role in subsequent intermediate metabolite degradation and dominated in the later stage of degradation. This successional change in microbial community structure has also been confirmed to be a crucial factor leading to the differences in the metabolic pathways of BaP (Patel et al., 2021).

The GO function annotations between groups are shown in Supplementary Figure S3, where annotated DEGs were most abundant in the categories of catalytic activity (GO:0003824), transporter protein activity (GO:0005215), structural molecule activity (GO:0005198), and metabolic process (GO:0008152) categories. At the initial stage of BaP stress (0–12 h), transporter activity with the highest proportion of gene abundance decreased from 54.2 to 30.29%, and the abundance change was relatively stable in the following 12–36 h. ABC transporter families have been reported to mediate the import and export of drugs and xenobiotics (Jeong et al., 2017). It was inferred that a large amount of BaP had been efficiently transported to the microorganisms during the initial stage of degradation, and the demand for large-scale transport of BaP was reduced in the later stage, so the abundance of transport proteins was maintained at a relatively stable level. Meanwhile, the abundance of catalytic activity genes increased significantly from 32.53 to 59.81% and then stabilized, which indicated that the transcription of genes encoding enzymes with potential catalytic activity was highly upregulated in the early stage of BaP degradation, potentially driving the oxidation, dehydrogenation and other reactions involved in this stage. In a study of the naphthalene-enhanced biodegradation of phenanthrene by *Pseudomonas* sp. SL-6 based on omics analysis, Cao et al. (2021) found that the categories significantly up-regulated in the results of genome annotation and differentially expressed protein analysis were mainly degradative enzymes, ABC transporter proteins, and electron transport carriers.

The degradation ability of PAHs degraders is related to the expression of their functional genes. The direct degradation of PAH by the degrader begins with the initial oxidation catalyzed by dioxygenase and then proceeds through the re-aromatization catalyzed by dehydrogenase, a process described as the upstream metabolic pathway for PAH degradation (Zhang et al., 2006). Xenobiotics biodegradation and metabolism of Kefir microorganisms in KEGG database annotation results metabolism mainly involves benzoate degradation (Ko00362), Aminobenzoate degradation (Ko00627), xylene degradation (Ko00622), degradation of polycyclic aromatic hydrocarbons (Ko00624), caprolactam degradation (Ko00930), drug metabolism - Cytochrome P450 (Ko00982) and drug metabolism-other enzymes (Ko00983), among others Table 4 shows some upstream functional genes involved in the degradation of BaP by Kefir microorganisms in these metabolic pathways. The metagenome was found to encode critical enzymes such as salicylate hydroxylase (EC 1.14.13.1) and the *α*/*β* subunits of protocatechuate 3,4-dioxygenase (EC 1.13.11.3). Through these enzymes, intermediates can be further metabolized by aromatic ring cleavage and the beta-ketoadipate pathway. Generally, the community may prefer oxygenase -mediated metabolism for degrade PAHs. It directly incorporates molecular oxygen at the ortho- or meta-sites of aromatic structures, exhibiting broad substrate specificity toward benzene derivatives, catechol, and biphenyls (Qiu et al., 2022; Rathour et al., 2021). Functioning as a mono-oxygenase, salicylic acid hydroxylase utilizes NADH or NADPH as an electron donor. This co-substrate facilitates the insertion of one oxygen atom into the target substrate, while the remaining oxygen atom is reduced to water (Liu et al., 2017). Protocatechuic 3,4-dioxygenase is a non-heme iron redox enzyme widely distributed in microorganisms that initiates the alienation degradation of aromaticity aromatic compounds through the β-ketoadipic acid pathway. The enzyme usually consists of a heterodimeric structure of α (*PcaG*) and β (*PcaH*) subunits (Frazee et al., 1993; Zylstra et al., 1989), where the α subunit specifically responsible for substrate binding (Ohlendorf et al., 1988).

**Table 4:** KO description of functional genes related to BaP degradation.

Gene	KO ID	Function
nidA	K11943	PAH dioxygenase large subunit [EC:1.13.11.-]
nidD	K11947	aldehyde dehydrogenase [EC:1.2.1.-]
[EC:1.14.13.1]	K00480	salicylate hydroxylase [EC:1.14.13.1]
pcaG	K00448	protocatechuate 3,4-dioxygenase, alpha subunit [EC:1.13.11.3]
pcaH	K00449	protocatechuate 3,4-dioxygenase, beta subunit [EC:1.13.11.3]
phdE	K18257	cis-3,4-dihydrophenanthrene-3,4-diol dehydrogenase [EC:1.3.1.49]
phdJ	K11949	4-(2-carboxyphenyl)-2-oxobut-3-enoate aldolase [EC:4.1.2.34]
dbfA1	K14599	dibenzofuran dioxygenase subunit alpha [EC:1.14.12.-]
flnB	K14601	1,1a-dihydroxy-1-hydro-9-fluorenone dehydrogenase
flnD1	K14602	2′-carboxy-2,3-dihydroxybiphenyl 1,2-dioxygenase large subunit
flnE	K14604	2-hydroxy-6-oxo-6-(2′-carboxyphenyl)-hexa-2,4-dienoate hydrolase [EC:3.7.1.-]
phtAa	K18251	phthalate 3,4-dioxygenase subunit alpha [EC:1.14.12.-]
phtC	K18256	3,4-dihydroxyphthalate decarboxylase [EC:4.1.1.69]
nahC	K14583	1,2-dihydroxynaphthalene dioxygenase [EC:1.13.11.56]
nahE	K14585	trans-o-hydroxybenzylidenepyruvate hydratase-aldolase [EC:4.1.2.45]
nahF	K00152	salicylaldehyde dehydrogenase [EC:1.2.1.65]
benB-xylY	K05550	benzoate/toluate 1,2-dioxygenase subunit beta [EC:1.14.12.10 1.14.12.-]
catA	K03381	catechol 1,2-dioxygenase [EC:1.13.11.1]
catB	K01856	muconate cycloisomerase [EC:5.5.1.1]
catC	K03464	muconolactone D-isomerase [EC:5.3.3.4]
CYP53A1	K07824	benzoate 4-monooxygenase [EC:1.14.14.92]
ligB	K04101	protocatechuate 4,5-dioxygenase, beta chain [EC:1.13.11.8]
nagX	K22270	3-hydroxybenzoate 6-monooxygenase [EC:1.14.13.24]
chqB	K04098	hydroxyquinol 1,2-dioxygenase [EC:1.13.11.37]
dmpL	K16243	phenol/toluene 2-monooxygenase (NADH) P1/A1 [EC:1.14.13.244 1.14.13.243]

### Prediction of the BaP degradation pathway

**Figure 4:**
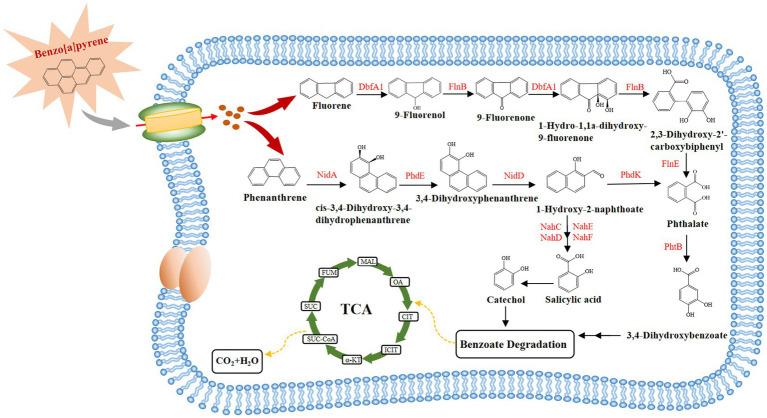
Predicted metabolic pathway and functional genes involved in BaP degradation by kefir microbiota. Black font: Substrate and intermediate product: Benzo[a]pyrene, Fluorene, Phenanthrene, 9-Fluorenol, 9-Fluorenone, 1-Hydro-1,1a-dihydroxy-9-fluorenone, 2,3-dihydroxy-2′-carboxybiphenyl, cis-3,4-Dihydroxy-3,4-dihydrophenanthrene, 3,4-Dihydroxyphenanthrene, 1-Hydroxy-2-naphthoate, Phthalate, 3,4-Dihydroxybenzoate, Catechol, Salicylic acid. Red font: Functional genes: D*bfA1*, *FlnB*, *NidA*, *PhdE*, *NidD*, *PhdK*, *FlnE*, *PhtB*, *NahC*, *NahE*, *NahD*, *NahF*. Black arrow: Metabolic pathway: salicylate and phthalate pathways.

Combined with the annotation of functional genes in the KEGG database (Table 4) and metabolic pathway map analysis, we can postulate potential biodegradation pathways for BaP degradation. As shown in Figure 4, it is postulated that after a series of initial transformations, the metabolic intermediates of BaP are directed into the downstream degradation pathways of phenanthrene and fluorene. Specifically, Kefir microorganisms degrade phenanthrene via two distinct routes. The first route involves the oxidation of 1-hydroxy-2-naphthoic acid to 2-dihydroxynaphthalene, which subsequently enters the naphthalene pathway to form salicylate for further catabolism. Alternatively, the aromatic ring of 1-hydroxy-2-naphthoic acid undergoes direct cleavage, directing the metabolites into the phthalate pathway. Supporting this metabolic divergence, a recent study demonstrated that a co-culture of *Rhodococcus* sp. WB9 and *Mycobacterium* sp. WY10 accelerates phenanthrene degradation through metabolic cross-feeding, utilizing both the salicylate and phthalate pathways (Sun et al., 2021). Furthermore, the degradation of fluorene proceeds via the formation of 2,3-dihydroxy-2′-carboxybiphenyl, catalyzed synergistically by dioxygenase and dehydrogenase, which subsequently directs metabolites into the phthalate pathway to facilitate significant BaP degradation. Overall, this analysis postulates that Kefir microorganisms degrade BaP through two primary routes: the salicylate and phthalate pathways. Corroborating this postulation, a study by Zhang et al. (2025) demonstrated that *Bacillus* sp. M1 similarly degrades BaP through both the salicylic acid and phthalic acid pathways. However, KEGG-based inference is inherently limited to cataloged pathways and may overlook novel organism-specific variants or emergent properties arising from microbial cross-feeding within the Kefir consortium. Therefore, emerging computational frameworks that reconstruct metabolic networks *de novo*, such as PathwaySeeker (bioRxiv, 2026), will be needed to uncover these unexplored metabolic pathways.

## Conclusion

Kefir microorganisms exhibited a strong ability to removal ability to BaP and possessed a relatively abundant set of genes associated with aromatic hydrocarbon degradation-related genes. PB testing screened out C (Na_2_HPO_4_ 2H_2_O), D (NaCl) and I (trace elements solution) as the key medium components. After response surface optimization, the degradation rate of BaP by Kefir reached 51.78% within 48 h under the following conditions: 12.50 mL of trace elements solution, 1.78 g/L Na_2_HPO_4_ 2H_2_O and 2.88 g/L NaCl. Meta-transcriptomic analysis identified genes encoding key degradation enzymes including salicylate hydroxylase and protocatechuate 3,4-dioxygenase, postulating the potential involvement of both salicylic acid and phthalic acid pathways in the kefir microbial degradation of BaP. Kefir microbiota as a natural complex microbial system with self-sustaining bioreactor characteristics, the kefir microbiota has potential for use in degrading BaP produced during high temperature food processing. Additionally, the identified kefir microorganisms, with its suggested degradation potential and associated genes, represents a potential source of strains for constructing food-grade microbial agents against PAHs.

## Data Availability

The datasets presented in this study can be found in online repositories. The names of the repository/repositories and accession number(s) can be found in the article/Supplementary material.

## Footnotes

1
http://www.geneontology.org/

2
https://www.genescloud.cn/
