# Supplementary material for: Benzo[a]pyrene degradation by Kefir-derived microbiota: medium optimization and metabolic pathways
Source: Front Microbiol. 2026 Jul 15;17:1873828. doi: 10.3389/fmicb.2026.1873828 (PMC13415574; doi:10.3389/fmicb.2026.1873828)
Supplement: Supplementary file 1 [file Table_1.DOCX]

Benzo[a]pyrene degradation by Kefir-derived microbiota: Medium optimization and metabolic pathways

Qing Wang^1†^, Yi Hu^1†^, Bei Zheng^1^, Zhuonan Yang^1^, Qianjin Lv^1^, Rui Zhang^1*^ and Yanan Qin^2*^

1 Xinjiang Key Laboratory of Special Species Conservation and Regulatory Biology, College of Life Science, Xinjiang Normal University, Urumqi 830054, Xinjiang, China

2 Xinjiang Key Laboratory of Biological Resources and Genetic Engineering, College of Life Science & Technology, Xinjiang University, Urumqi 830046, Xinjiang, China

^†^ Q.W. and Z.N.Y. contributed equally to this work.

*Corresponding Author

Prof. Rui Zhang

Email: [zhangrui1124@xjnu.edu.cn,](mailto:zhangrui1124@xjnu.edu.cn,)

Phone:15209913105

Supplementary Information: 4 pages, 3 tables, 3 figures

Table S1 Factors and levels of Plackett-Burman experiment design

| Code | Factor | g·L^-1^/mL·L^-1^ Level | |
| --- | --- | --- | --- |
|  |  | -1 | +1 |
| A | KH_2_PO_4_ | 0.2 | 2 |
| B | K_2_HPO_4_ | 0.4 | 4 |
| C | Na_2_HPO_4_ 2H_2_O | 0.3 | 3 |
| D | NaCl | 0.5 | 5 |
| E | NH_4_Cl | 0.4 | 4 |
| F | MgSO_4_ 6H_2_O | 0.1 | 0.3 |
| G | MnSO_4_ | 0.01 | 0.1 |
| H | FeCl_3_ | 0.01 | 0.03 |
| I | Trace Elements Solution SL-4 | 5mL | 25mL |

(Trace Elements Solution SL-4: EDTA 0.5 g, CoCl_2_·6H_2_O 0.02 g, FeSO_4_·7H_2_O 0.2 g, CuCl_2_·2H_2_O 0.001 g, ZnSO_4_·7H_2_O 0.01 g, NiCl_2_·6H_2_O 0.002 g, MnCl_2_·4H_2_O 0.003 g, Na_2_MoO_4_·2H_2_O 0.003 g, H_3_BO_3_ 0.03 g, Distilled water 1,000.0 mL, pH 3.4)

Table S2 Plackett-Burman design matrix indicating media composition used in various runs and the results on % BaP degradation

| code | A | B | C | D | E | F | G | H | I | degradation rate(%) |
| --- | --- | --- | --- | --- | --- | --- | --- | --- | --- | --- |
| 1 | 1 | -1 | -1 | -1 | 1 | -1 | 1 | 1 | -1 | 31.2 |
| 2 | 1 | 1 | -1 | 1 | 1 | 1 | -1 | -1 | -1 | 34.79 |
| 3 | -1 | 1 | 1 | 1 | -1 | -1 | -1 | 1 | -1 | 0 |
| 4 | 1 | -1 | 1 | 1 | -1 | 1 | 1 | 1 | -1 | 4.23 |
| 5 | -1 | 1 | 1 | -1 | 1 | 1 | 1 | -1 | -1 | 25.06 |
| 6 | -1 | -1 | -1 | -1 | -1 | -1 | -1 | -1 | -1 | 19.62 |
| 7 | 1 | -1 | 1 | 1 | 1 | -1 | -1 | -1 | 1 | 23.80 |
| 8 | 1 | 1 | -1 | -1 | -1 | 1 | -1 | 1 | 1 | 48.78 |
| 9 | 1 | 1 | 1 | -1 | -1 | -1 | 1 | -1 | 1 | 32.79 |
| 10 | -1 | -1 | -1 | 1 | -1 | 1 | 1 | -1 | 1 | 22.72 |
| 11 | -1 | 1 | -1 | 1 | 1 | -1 | 1 | 1 | 1 | 38.27 |
| 12 | -1 | -1 | 1 | -1 | 1 | 1 | -1 | 1 | 1 | 33.85 |

Table S3 The response value of the dependent variable in relation to cyfluthrin degradation was determined using a Box-Behnken experimental design

| Number | I:Trace Elements Solution SL-4(mL) | C:Na_2_HPO_4_ 2H_2_O(g/L) | D:NaCl(g/L) | degradation rate (%) |
| --- | --- | --- | --- | --- |
| 1 | 20 | 3 | 3 | 26.78 |
| 2 | 20 | 2 | 5 | 30.98 |
| 3 | 15 | 3 | 1 | 27.32 |
| 4 | 20 | 1 | 3 | 29.67 |
| 5 | 15 | 2 | 3 | 46.81 |
| 6 | 10 | 2 | 1 | 39.56 |
| 7 | 10 | 2 | 5 | 33.77 |
| 8 | 15 | 2 | 3 | 52.83 |
| 9 | 10 | 3 | 3 | 36.43 |
| 10 | 15 | 1 | 5 | 33.17 |
| 11 | 15 | 2 | 3 | 49.08 |
| 12 | 10 | 1 | 3 | 46.72 |
| 13 | 15 | 2 | 3 | 51.91 |
| 14 | 15 | 3 | 5 | 27.16 |
| 15 | 20 | 2 | 1 | 28.29 |
| 16 | 15 | 2 | 3 | 48.27 |
| 17 | 15 | 1 | 1 | 32.72 |


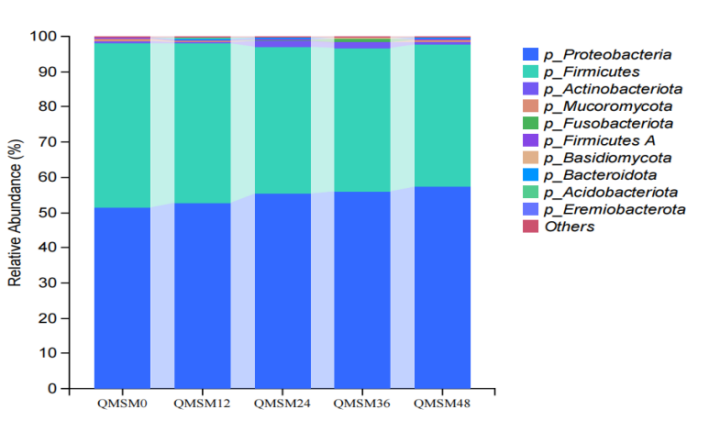


Figure S1. Microbial community bar plot of phylum.The relative abundance of microbial

communities at the phylum level in kefir samples.


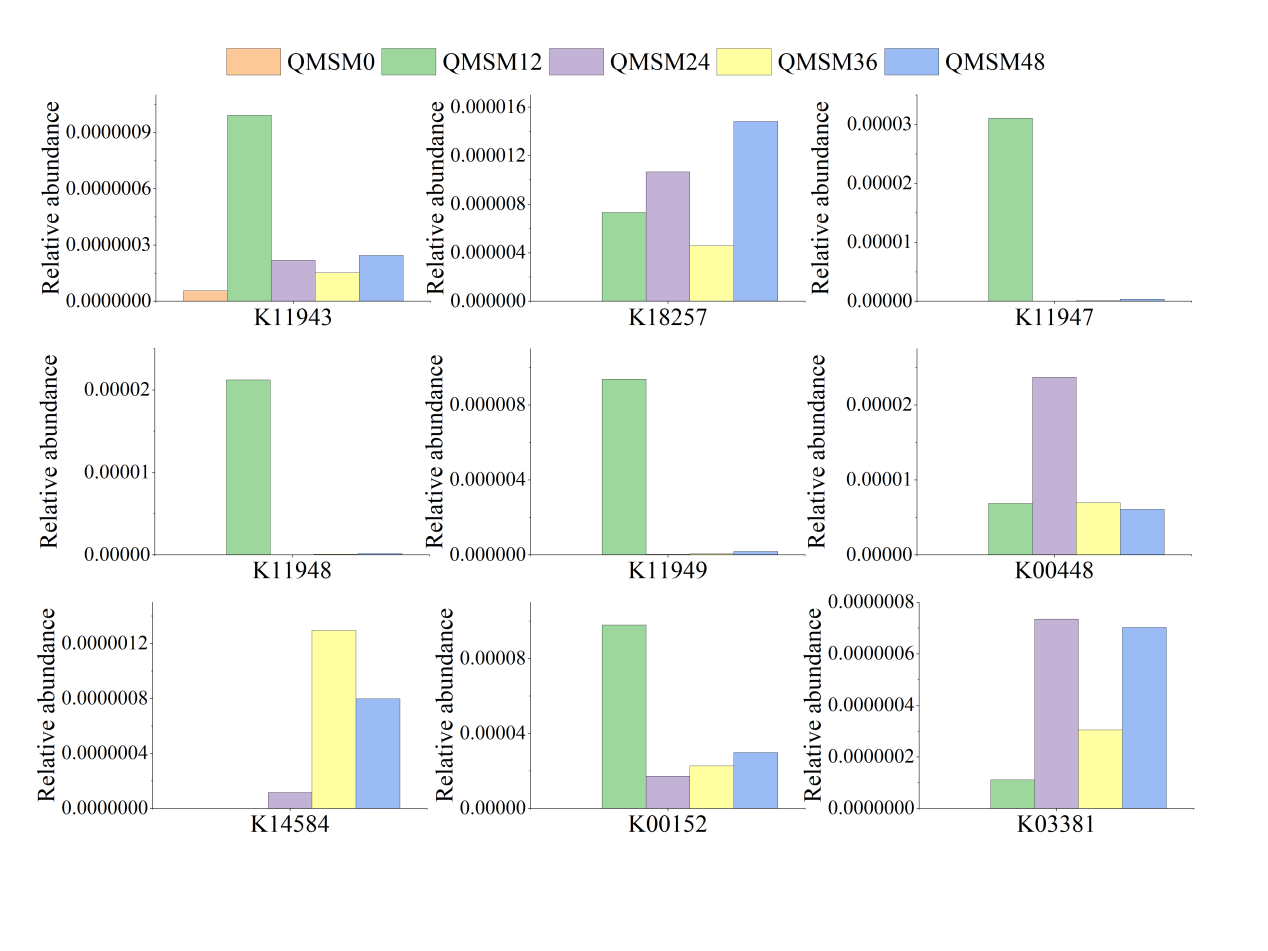


Figure S2. The relative abundance of some functional genes associated with BaP degradation, which were enriched by the kefir microbial community during BaP degradation under optimized medium composition conditions.


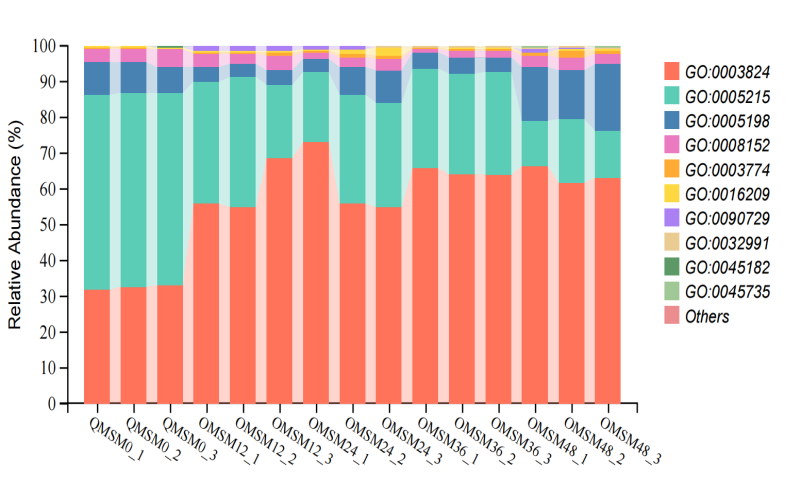


Figure S3. Gene Ontology (GO) functional annotation among groups. The annotated differentially expressed genes (DEGs) were predominantly enriched in the categories of catalytic activity (GO:0003824), transporter activity (GO:0005215), structural molecule activity (GO:0005198), and metabolic process (GO:0008152).
